# Yinchen Linggui Zhugan decoction ameliorates high fat diet-induced nonalcoholic fatty liver disease by modulation of SIRT1/Nrf2 signaling pathway and gut microbiota

**DOI:** 10.3389/fmicb.2022.1001778

**Published:** 2022-12-12

**Authors:** Hui Jiang, Tangyou Mao, Zhongmei Sun, Lei Shi, Xiao Han, Yang Zhang, Xiaosi Zhang, Jiali Wang, Juncong Hu, Liming Zhang, Junxiang Li, Haixiao Han

**Affiliations:** ^1^School of Graduate, Beijing University of Chinese Medicine, Beijing, China; ^2^Dongfang Hospital, Beijing University of Chinese Medicine, Beijing, China

**Keywords:** nonalcoholic fatty liver disease, Yinchen Linggui Zhugan decoction, gut microbiota, metabolites, short-chain fatty acids, spearman rank correlation analysis

## Abstract

Yinchen Linggui Zhugan decoction (YLZD) is an effective and classical traditional herbal prescription for treating the nonalcoholic fatty liver disease (NAFLD) and has been proven to be effective in the regulation of lipid metabolism disorder and attenuate inflammation for a NAFLD rat model. However, the exact underlying mechanism has not been elucidated. In the current study, a NAFLD rat model was established using a high-fat diet (HFD) for 10 weeks, followed by YLZD treatment with 1.92 g/kg/day for 4 weeks to explore the mechanisms of YLZD. Our results showed that YLZD decreased the hepatic lipid deposition, restored the liver tissue pathological lesions, inhibited the expression of oxidative stress, and decreased the inflammatory cytokines levels. Meanwhile, the genes and proteins expressions of SIRT1/Nrf2 signaling pathway together with downstream factors including HO-1 and NQO1 were elevated in the YLZD treated NAFLD rats. For further elaborating the upstream mechanism, short-chain fatty acids (SCFAs) in serum and feces were measured by liquid chromatograph mass spectrometer and gas chromatograph mass spectrometer, and the differences in gut microbiota of rats in each group were analyzed through high-throughput sequencing of 16S rRNA. The results demonstrated that the contents of butyric acid (BA) and total SCFAs in YLZD-treated NAFLD rats were significantly increased in serum and feces. 16S rRNA sequencing analysis illustrated that YLZD intervention led to a modification of the gut microbiota composition, with a decrease of *Oribacterium*, *Lactobacillus* and the ratio of *Firmicutes/Bacteroides*, as well as the increase in SCFAs-producing bacteria such as *Christensenellaceae*, *Clostridia*, *Muribaculaceae*, and *Prevotellaceae*. Spearman rank correlation analysis indicated that BA and total SCFAs were negatively co-related with oxidative stress-related factors and inflammatory cytokines, while they were positively co-related with SIRT1/Nrf2 pathway related genes and proteins. Furthermore, *in vitro* study confirmed that BA effectively reduced oxidative stress by activating SIRT1/Nrf2 signaling pathway in L02 cells. Together, the present data revealed YLZD could ameliorate HFD-induced NAFLD in rats by the modulation of SIRT1/Nrf2 signaling pathway and gut microbiota.

## Introduction

Nonalcoholic fatty liver disease (NAFLD) is a clinicopathological syndrome characterized by excessive fat deposition in hepatocytes and is an acquired metabolic stress liver injury (Hong et al., 2021). It has become a major causative factor for chronic liver disease with the globalization of obesity and metabolic syndrome. About one quarter of adults worldwide suffer from NAFLD, and the prevalence is even higher in Asian populations, which is about 27.37% (Younossi et al., 2016; Araújo et al., 2018). Worse still, no effective treatment or approved drug therapy for NAFLD is available at present. Currently, the NAFLD treatment primarily focuses on lifestyle improvements such as diet modification and weight control, but this is difficult to achieve and maintain in most of the patients (Ferguson and Finck, 2021). The pathogenesis of NAFLD is still unclear and it is widely believed that oxidative stress plays a pivotal role in the development of NAFLD (Hong et al., 2021). It has been documented that oxidative stress can lead to cellular dysfunction, damage, and ultimately cell death, and the impairment of antioxidant status in liver significantly results in the onset and progression of nonalcoholic liver disease (Arroyave-Ospina et al., 2021). In addition, gut microbiota and its metabolites were also considered to act a vital role in the pathophysiology of NAFLD (Aron-Wisnewsky et al., 2020).

Silencing information regulator 2 related enzyme 1 (sirtuin1, SIRT1) is a nicotinamide adenine dinucleotide (NAD^+^)-dependent deacetylase, which has been reported to be involved in the hepatic lipid metabolism regulation (Majeed et al., 2021), oxidative stress (Chung et al., 2021) and inflammatory response (Yang et al., 2022). Nuclear erythroid 2-related factor 2 (Nrf2) is a pivotal redox reactive transcription factor that regulates the expression of a range of antioxidant proteins. Nrf2 in the cytoplasm binds to Keap1 in an inhibitory state under physiological conditions. While it would be isolated from Keap1 and translocated to the nucleus as stimulated by oxidative stress signals, which activates antioxidant response elements (ARE) and up-regulates the expression of antioxidant factors such as heme oxygenase-1 (HO-1) and NAD (P) H: quinone oxidoreductase 1 (NQO1; Zhang et al., 2013). Besides, SIRT1 could not only activate Nrf2 and antioxidant genes by altering the structure of Keap1, but also directly promotes the deacetylation and subsequent activation of Nrf2, resulting in the upregulation of Nrf2 downstream genes to enhance the antioxidant capacity of the host (Huang et al., 2015; Kopacz et al., 2020). The above findings suggested that SIRT1 could stimulate Nrf2 and regulate the expression of antioxidant factors, which in turn acts as an antioxidant against oxidative stress damage.

In recent years, more metabolism-related diseases (including NAFLD) have been confirmed to be closely associated with dysbiosis of the gut microbiota with the change in people’s lifestyles (Aron-Wisnewsky et al., 2020; Dai et al., 2020). Short-chain fatty acids (SCFAs) are the main metabolites of gut microbiota. It has been suggested that SCFAs are vital for host metabolism and they are substrates for energy production, lipogenesis, gluconeogenesis and cholesterol synthesis (Bergman, 1990; den Besten et al., 2013). In addition, SCFAs could mediate lipid metabolism, inflammatory, and oxidative stress of host (Ding et al., 2019; Dai et al., 2020). Hence, it is of great importance to investigate the mechanism of gut microbiota and its metabolites participating in oxidative stress and lipid metabolism of NAFLD by targeting SCFAs.

Traditional Chinese medicine (TCM) was characterized by multiple pathways and multiple targets, with significant clinical efficacy and high safety. It had been confirmed that TCM could address the complex etiology and clinical manifestations of NAFLD (Xu et al., 2020; Du et al., 2021). Yinchen Linggui Zhugan decoction (YLZD) is a combination of the classic formulas Yinchenhao decoction and Linggui zhugan decoction from the Treatise on febrile diseases. It is widely used for treating the hepatobiliary diseases (Chen et al., 2021; Shi et al., 2021) and metabolic syndrome (Yao et al., 2017). Our previous research found that YLZD could effectively improve liver function, reduce blood lipid levels, and modify NAFLD-related pathological processes such as cellular autophagy (Guo et al., 2017). However, the molecular mechanisms of YLZD treating NAFLD have not been clearly elucidated. Accordingly, this study was carried out to investigate the effects of YLZD on the oxidative stress, elucidate the underlying mechanism of the protective effect of YLZD in the treatment of HFD-induced NAFLD through measuring SIRT1/Nrf2 signaling pathway. The differences in gut microbiota and SCFAs concentration between the model and treatment groups were analyzed by 16S rRNA sequencing, liquid chromatograph mass spectrometer (LC–MS) and gas chromatograph mass spectrometer (GC–MS). Furthermore, *in vitro* studies confirmed that BA can improve FFA induced oxidative stress in L02 cells by positively regulating SIRT1/Nrf2 signaling pathway. The results indicated that YLZD could alleviate the HFD-induced NAFLD *via* modulating gut microbiota composition and SCFAs concentration.

## Materials and methods

### Experimental animal

Twenty-four SPF-grade male Sprague–Dawley (SD) rats with the weight of 180 ± 20 g were bought from Beijing Vital River Laboratory Animal Technology Co., Ltd. (Beijing, China). All rats were housed in the Experimental Animal Center of Beijing University of Chinese Medicine under typical environmental surroundings at 22 ± 2°C, 50–60% relative humidity and 12 h light–dark. The rats had free access to standard laboratory food and water and the experiments were carried out after 1 week of adaptative feeding. The study protocols were reviewed and approved by the Animal Research Ethics Committee of Beijing University of Chinese Medicine (no. BUCM-4-2021072002-3151).

### Experimental drugs

The composition and corresponding dosage of YLZD base drug are as follows: *Artemisiae scopariae* 18 g (formula granules: 1.8 g), *Gardeniae fructus* 9 g (formula granules: 0.9 g), *Radix rhei et rhizome* 6 g (formula granules: 1.2 g), *Poria* 12 g (formula granules: 2.4 g), *Cinnamomi ramulus* 9 g (formula granules: 0.45 g), *Atractylodes macrocephala koidz* 6 g (formula granules: 1.2 g), and *Licorice* 6 g (formula granules: 1.2 g). The YLZD formula granules used in this study were purchased from the Department of Pharmacy, Dongfang Hospital, Beijing University of Chinese Medicine (Beijing, China). The formulation granule process is made by Beijing Tcmages Pharmaceutical Co., Ltd. (Beijing, China) using Chinese herbal medicine as raw materials through the steps of extraction, separation, concentration, drying, granulation and packaging. The main components of YLZD formula granules were identified as chlorogenic acid, geniposide, isochlorogenic acid B, isochlorogenic acid A, isochlorogenic acid C, aloe-emodin, rhein, glycyrrhizic acid, and emodin by High Performance Liquid Chromatography (HPLC) with the specific corresponding contents of 2.8521, 11.8532, 0.6459, 0.3511, 0.8627, 0.0971, 0.1098, 2.9140, and 0.0088 (mg/g), respectively (Jiang et al., 2022).

### Preparation and intervention of NAFLD rat model

Twenty-four SD rats were randomly divided into a negative control group (Control, *n* = 9) and a model group (*n* = 15). These two groups were fed with standard laboratory feed and high-fat diet (HFD) feed for 10 weeks, respectively. The HFD was provided by SPF (Beijing) BIOTECHNOLOGY Co., Ltd. (Beijing, China) and consisted of 88% basic feed, 10% lard and 2% cholesterol. At the end of the 10th week, three rats in the Control and model groups, respectively, were randomly sacrificed and the liver histopathology staining results verified that NAFLD rats were successfully modeled. The remaining twelve model group rats were randomly divided into HFD group and YLZD group, with 6 rats in each group.

The YLZD granules were dissolved in water (0.192 g/ml) at 80°C, cooled to room temperature and then gavaged. The dose of YLZD gavage was 10 ml/kg/day, which was the optimal intervention dose screened in our previous study (Jiang et al., 2022). Control and HFD group were gavaged with sterile distilled water (10 ml/kg). The drug or sterile distilled water was administered once a day at a relatively fixed time (15–17 p.m.) for 4 consecutive weeks. The body weight and food intake of each rat were recorded regularly every week. On the day of feces sampling (1 day before the end of the experiment), the sterile bedding was changed and the rats were fed separately. Rat feces were taken with alcohol-sterilized forceps, which were fully disinfected with alcohol wipe before clamping another rat’s feces. The collected feces were placed into sterile EP tubes, and were quickly placed in liquid nitrogen for freezing. After the last administration, all rats were fasted for 24 h and the blood samples were taken from the abdominal aortic under anesthesia. Excised fresh liver tissues were immersed in a 4% paraformaldehyde solution or rapidly frozen in liquid nitrogen. All specimens were stored in a refrigerator at −80°C until analysis.

### Histological analysis

Fresh liver tissues from the same part of the liver for each rat were fixed in 4% paraformaldehyde solution for 24 h. The liver tissue samples were then paraffin embedded, histologically sectioned (5 μm), stained with hematoxylin and eosin (HE), and photographed through a light microscope. Moreover, the frozen liver tissues were cut into sections (10 μm) and stained with oil red O (ORO) and the distribution of lipid droplets in liver tissues were characterized using a light microscopy.

### Enzyme linked immunosorbent assay

One gram frozen liver tissue was added with 9 ml phosphate buffer (PH 7.2–7.4, 0.01 mmol/l), then the mixture was homogenized and centrifuged (3,000 ×*g*, 4°C, 20 min) to obtain a homogenate. The free fatty acids (FFA, No. YJ780232), total cholesterol (TC, No. YJ789922), triglyceride (TG, No. YJ892022), total antioxidant capacity (T-AOC, No. YJ067823), reactive oxygen species (ROS, No. YJ987634), malondialdehyde (MDA, YJ543672), superoxide dismutase (SOD, YJ098743) and glutathione peroxidase (GSH-Px, YJ652893) were detected according to the kit instructions. In addition, the whole blood samples were placed at room temperature for 2 h and centrifuged at 3000 rpm for 15 min at 4°C, and TNF-α (No. 88–7,340-77), IL-6 (No. ERA31RB) together with IL-1β (No. EK301B/3–02) levels in the supernatant were detected followed by the kit instructions. The kits were provided by Invitrogen, MultiSciences (Lianke) Biotech Co., Ltd. (Hangzhou, China) and Shanghai Enzyme-linked Biotechnology Co., Ltd. (Shanghai, China).

### Immunohistochemistry assay

Immunohistochemistry (IHC) was adopted to localize and quantify SIRT1 and Nrf2 proteins in liver tissues. In brief, the tissue sections were dewaxed and hydrated with xylene and gradient alcohol. Citric acid antigen repair buffer (PH 6.0, No: G1202, Servicebio, Wuhan, China) was used for antigen repair. Peroxidase was removed by incubation with 0.3% hydrogen peroxide at room temperature for 10 min, and non-specific staining was reduced by incubation with super blocking solution for 5 min. The primary antibodies of SIRT1 (1, 1,000, No. GB113808) and Nrf2 (1, 1,000, No. GB11171) were added dropwise and incubated overnight at 4°C. The above antibodies were provided by Proteintech Group, Inc. Wuhan, China. Then the HPR labeled goat anti-rabbit secondary antibody (1, 200, No. GB23303, Servicebio, Wuhan, China) was added and incubated at room temperature for 50 min. Optical density was measured by Image-Pro Plus 6.0 (Media Cybernetics, Bethesda, MD, United States). Integrated optical density (IOD)/area represents the positive staining area and intensity of a specific protein per unit area (Liao et al., 2020).

### Real time quantitative PCR analysis

Total RNA was extracted from liver tissues by adding TRIzol reagent (No: DP424, TIANGEN BIOTECH (BEIJING) CO., LTD, Beijing, China) and chloroform. The concentration and purity of RNA were determined by NanoDrop® Nd-2000 (ThermoFisher Scientific). cDNA reverse transcription was performed *via* the PrimeScript™ RT reagent Kit with gDNA Eraser (No: RR047B, TaKaRa, Beijing, China). The mRNA expression levels of *SIRT1*, *Nrf2*, *HO-1* and *NQO-1* in liver tissues were detected by real time quantitative PCR (RT-qPCR) amplification with *ACTIN* as internal reference. Primers were designed and synthesized by Invitrogen and their sequences were given in Table 1. PCR reaction system: 2 × Master Mix 10 μl, forward primer 0.5 μl, reverse primer 0.5 μl, cDNA 2 μl, and DEPC water 7 μl. Reaction conditions: predenaturation at 95°C for 30 s, denaturation at 95°C for 15 s, annealing and extension at 60°C for 1 min, and repeat the above steps for 40 cycles. The relative expression of target gene was measured by 2^-ΔΔCt^ method.

**Table 1:** Primer sequences used for RT-qPCR.

Gene names	Accession number	Forward primer (5′ → 3′)	Reverse primer (5′ → 3′)
Silencing information regulator 2 related enzyme 1 (*SIRT1*)	NM_001372090	CATAGGTTAGGTGGCGAGTA	GTGTAGCAATCACAGAAGAG
Nuclear erythroid 2-related factor 2 (*Nrf2*)	NM_001399173	AAGATGCCTTGTACTTTGAAGACTGT	GGAAAATAGCTCCTGCCAAACTT
Heme oxygenase-1 (*HO-1*)	NM_012580	AGCACAGGGTGACAGAAGAG	AACTCTGTCTGTGAGGGACT
NAD(P)H:quinone oxidoreductase 1 (*NQO-1*)	NM_017000	GAAGAAGCGTCTGGAGACTGTC	CGGCTGGAATGGACTTGC
*ACTIN*	J02781	AGAGGGAAATCGTGCGTGA	CATTGCCGATAGTGATGACCT

### Serum and Fecal SCFAs quantitative analysis

Liquid chromatograph mass spectrometer (LC–MS) was adopted to determine the content of serum SCFAs. In brief, a total of 50 μl serum sample was added to 100 μl of acetonitrile extract, and the supernatant was extracted after 30 min of low-temperature sonication (5°C, 40 KHz) and centrifugation (4°C, 13, 000 ×*g*, 15 min). 20 μl, 200 mM 3NPH.HCL and 20 μl 120 mM EDC.HCL (containing 6% pyridine) were added to the supernatant at 40°C for 30 min, and finally the solutions were diluted with 50% aqueous acetonitrile to 750 μl for machine detection. The concentration of acetic acid (AA), propionic acid (PA), butyric acid (BA), isobutyric acid (IBA), valeric acid (VA), isovaleric acid (IVA), hexanoic acid (HA), and isohexanoic acid (IHA) were determined by LC-ESI-MS/MS (UHPLC-QTRAP) (AB Sciex Pte. Ltd.) equipped with a Waters BEH C18 (150 × 2.1 mm, 1.7 um) liquid chromatographic column. The mobile phase A and B were 0.1% formic acid-water solution and 0.1% formic acid-acetonitrile, respectively, the flow rate was 2.0 μl/min and the column temperature was 40°C. The AB Sciex quantitative software OS was used to automatically identify and integrate the ion fragments. The linear regression standard curve was plotted using the mass spectral peak area and concentration of the analyte as the vertical coordinate and horizontal coordinate, respectively. The mass spectral peak areas of the analytes were substituted into the linear equation to calculate the concentrations. SCFA (ng/ml) = (C × DF × V)/W, where C means the concentration measured by LC–MS, V presents the volume of the fixed volume, DF is the dilution, and W is the sampling volume.

The content of fecal SCFAs was determined by a gas chromatograph mass spectrometer (GC–MS). In short, a total of 25 mg fecal samples was homogenized with 500 μl water (containing 0.5% phosphoric acid) for 3 min (50 HZ, twice), then it was centrifuged at 13, 000 ×*g* for 15 min. After that, 0.2 ml n-butanol solvent (containing 10 μg/ml of internal standard 2-ethylbutyric acid) was added, followed by low-temperature sonicate for 10 min and centrifugation. The concentration of fecal SCFAs was determined by 8890B-7000D GC/MSD (Agilent Technologies Inc., CA, United States) equipped with a HP FFAP capillary column (30 m × 0.25 mm × 0.25 μm). High purity helium (purity not less than 99.999%) was used as carrier gas, the flow rate was 1.0 ml/min and the inlet temperature of 260°C. The ionic fragments of the target SCFAs were automatically identified and integrated *via* mass-hunter quantification software (Agilent inc., United States, v10.0.707.0). The concentration of the target in the sample is calculated by substituting the peak area of the target into the linear equation of the standard curve, and the actual target content is calculated based on the sampling volume. SCFA (μg/mg) = (C × V)/M, where C means the concentration determined by GC–MS on board, V presents the fixed volume, and M is the sample weight.

### DNA extraction, 16S rRNA gene sequencing

E.Z.N.A. Stool DNA kit (Omega Bio-tek, Norcross, GA, United States) was performed to extract the microbial DNA in fecal samples (200 mg). The DNA samples were amplified by PCR in the V3-V4 (338F_806R) region of 16S ribosomal RNA gene after the quality test. Primers 338F and 806R are ACTCCTACGGGAGGCAGCAG and GGACTACHVGGGTWTCTAAT, respectively. The amplification was purified by a AxyPrep DNA Gel Extraction Kit (Axygen Biosciences, Union City, CA, United States) and was quantified *via* QuantiFluor™-ST (Promega, United States). Illumina MiSeq platform (Illumina, San Diego, CA, United States) was employed to sequence. The raw fastq files were quality filtered by Trimmomatic and merged by FLASH according to the criteria used in the previous research (Sun et al., 2020). Then, the quality of Reads was controlled and filtered for the sequences obtained by Illumina Misep sequencing, and these sequences were clustered into operational taxonomic units (OTUs) with a 97% sequence identity using Usearch (version 11.0) and Uparse (version 7.0.1090). Next, the Alpha diversity index (Chao richness and Shannon diversities) was calculated through Mothur (version 1.30.2). Principal coordinate analysis (PCoA) on the basics of Bray–Curtis dissimilarity was carried out using the R package (version 3.3.1) for illustrating the clustering of different samples. Community Bar maps were generated using R software based on the species annotation results of OTUs to show the dominant species at the Phylum and Genus levels for each group of samples and the relative abundance (proportion) of each dominant species. The differences in dominant bacterial community between groups was determined by Kruskal-Wallis H.

### Cell culture and treatment

L02 cells were purchased from Shanghai Fuheng Biotechnology Co., Ltd., and cultured in RPMI-1640 (No. SH30027, Hyclone) supplemented with 10% fetal bovine serum (FBS, No. SH30084.03, Hyclone), 100 U/ml penicillin and streptomycin in an incubator with 5% CO2 and high relative saturation humidity (95%) at 37°C. L02 cells were exposed to an FFA mixture (Oleic acid: Palmitoleic acid at 2: 1) at a final concentration of 1 mM for 24 h to construct NAFLD cell model. And then the cells were treated with AA (1.0 mM), BA (0.5 mM) and EX-527 (an SIRT1 inhibitor, 100 nM) for 24 h. The L02 cells cultured in a medium without other drugs were used as control. Oleic acid (No. HY-N1446B) and EX-527 (No. HY-15452) were supplied by MedChemExpress (Shanghai, China), and palmitoleic acid (No. S161420), AA (No. A298827) together with BA (No. S102956) were purchased from Aladdin Holding Group Co., Ltd. (Beijing, China).

### Cell viability assay

L02 cells were plated at a density of 7 × 10^3^ cells per well into a 96-well plates to full confluence for 24 h, and then treated with various concentrations of FFA (0, 0.125, 0.25, 0.5, 1, 1.5, 2 mM), AA (0, 0.1, 0.5, 1, 2.5, 5, 10, 20 mM), BA (0, 0.01, 0.05, 0.1, 0.5, 1, 5, 10 mM) and EX-527 (0, 50, 100, 200, 500, 1,000 nM) for 24 h, respectively. Cell Counting Kit-8 (CCK8, DOJINDO LABORATORIES, Shanghai, China) was used to estimate cell viability in strict accordance with the specification.

### Oil red O staining *in vitro*

L02 cells were plated at a density of 4 × 10^5^ cells per well into a six-well plates and exposed to FFA with or without AA, BA, or EX-527 for 24 h. After fixation with 4% paraformaldehyde for 30 min at room temperature, the fixed cells were stained in ORO solution for 30 min, and then their nucleus was stained by hematoxylin for 3 min. The cell culture plates were placed under the microscope to evaluate the effect of lipogenic staining. Quantitative analysis of ORO staining results was performed using Image Pro Plus 6.0 software (Media Cybernetics, Inc., Rockville, MD, United States).

### Determination of intracellular reactive oxygen species

The intracellular reactive oxygen species (ROS) levels were measured by the ROS assay kit (No. CA1410, Solarbio, China) through the conversion of a DCFH-DA fluorescence probe. In brief, L02 cells were exposed to FFA (1 mM) for 24 h and then treated with BA or EX-527 for 24 h. Subsequently, the cells were incubated with an appropriate amount of DCFH-DA (10 μM/l) for 30 min at 37°C. Finally, the DCF fluorescence intensity was analyzed by a flow cytometer (Agilent, United States).

### Biochemical analysis

The cultured L02 cells were trypsinized and collected for further analysis. In short, L02 cells were collected into a centrifuge tube and added appropriate volume of extracting solution (1 ml/500 × 10^4^ cells). Then the cells were sonicated for 1 min, centrifuged for 10 min (8,000 ×*g*, 4°C) and removed the supernatant for testing. The intracellular TG (No. BC0625), SOD (No. BC0170), MDA (No. BC0020), and GSH-Px (No. BC1190) were performed according to the kit instructions. The commercially available kits were provided by Beijing Solarbio Science & Technology Co., Ltd. (Beijing, China).

### Western blot analysis

Total proteins were extracted from liver tissues and cells using RIPA buffer (No: G2002, Servicebio, Wuhan, China) supplemented with protease and phosphatase inhibitors. Protein concentration was measured with BCA protein concentration assay kit (No: G2026, Servicebio, Wuhan, China). Then, equal amounts of protein were separated by 8–10% SDS-polyacrylamide gel followed by transfering to 0.45 μm polyvinylidene difluoride (PVDF) membranes (No: G6015-0.45, Servicebio, Wuhan, China). The PVDF membranes were blocked in TBST with 5% nonfat dry milk, and then incubated overnight with primary antibodies against SIRT1 (1, 2000, No. 16396-1-ap) and Nrf2 (1, 3,000, No. 13161-1-ap) (Proteintech Group, Inc. Wuhan, China). Secondary antibody diluted with TBST (1, 5,000) was added and incubated for 30 min at room temperature. Finally, signals were visualized by enhanced chemiluminescence detection.

### Statistical analysis

Data was statistically analyzed by GraphPad Prism 8.0 (La Jolla, CA, United States). The measurement data of each group was expressed as mean ± standard error of the mean (SEM). Multiple group comparisons were performed using one-way analysis of variance (ANOVA) followed by Tukey’s multiple comparison test. Spearman rank correlation analysis was performed for correlation analysis and the results were considered significant at *p* < 0.05.

## Results

### YLZD improved the general status and mitigated hepatic lipid deposition

**Figure 1:**
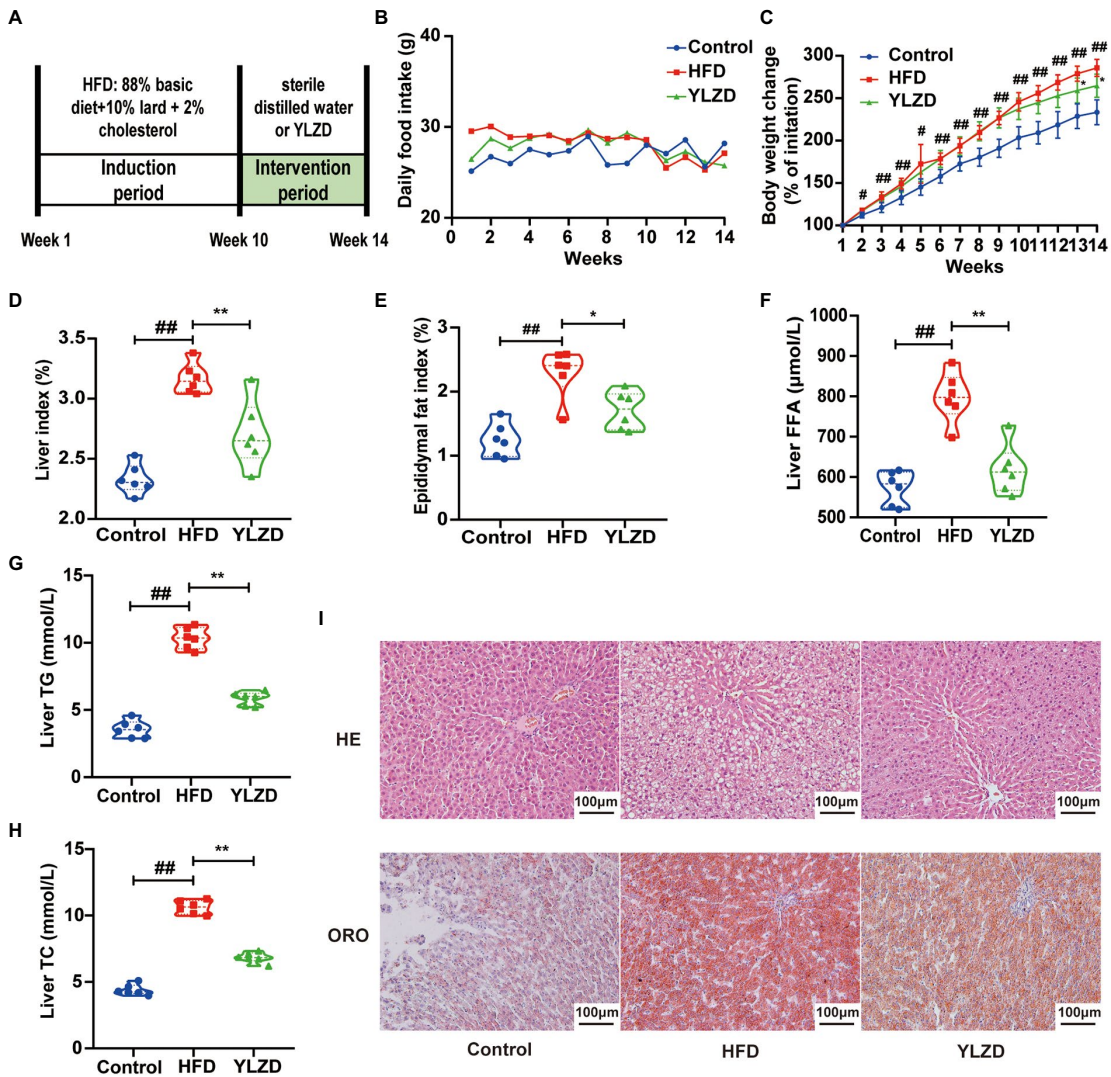
YLZD improved the general status and mitigated hepatic lipid deposition. **(A)** Experimental design, **(B)** daily food intake, **(C)** body weight change, **(D)** Liver index = Liver weight/Body weight × 100, **(E)** Epididymal fat index = Epididymal fat weight/Body weight × 100, **(F–H)** The level of liver FFA, TG and TC, respectively, and **(I)** Representative images of HE and oil red staining livers (magnification, ×200). Control group, normal rats treated with distilled water; HFD group, HFD-induced liver injury rats treated with distilled water; YLZD group, HFD induced liver-damaged rats treated with YLZD 1.92 g/kg/day for 4 weeks. Values are represented as mean ± SEM (*n* = 6 rats/group). ^##^*p* < 0.01, ^#^*p* < 0.05 versus the Control group; ^**^p < 0.01, ^*^p < 0.05 versus the HFD group.

HFD-induced NAFLD animal models are characterized by high stability and low cost, and are closer to the pathological and molecular mechanisms of human NAFLD (Kanuri and Bergheim, 2013). In present study, NAFLD rat model was established by HFD feeding for 10 weeks. Liver histopathological staining results showed a significant steatosis and ballooning of the liver tissue in the model group rats compared with the Control group rats, indicating that a rat model of NAFLD was successfully established by the 10-week HFD diet (Supplementary Figure S1). Subsequently, the NAFLD rats were intervened by YLZD with the dose of 1.92 g/kg/day for 4 weeks. The experimental flow chart was shown in Figure 1A. It is readily apparent that no significant difference in food intake between the different group of rats was observed throughout the experimental period (Figure 1B). At the end of the experiment, the body weight, liver and epididymal fat index of the rats in HFD group were dramatically higher than those for Control group (*p* < 0.05 or 0.01, Figures 1C–E). Meanwhile, YLZD significantly decreased the body weight, liver and epididymal fat index (*p* < 0.05 or 0.01, Figures 1C–E). In addition, the liver FFA, TG, and TC levels in HFD group were distinctly increased (*p* < 0.01, Figures 1F–H) by comparison with the Control group. YLZD treatment effectively reduced these indicator levels (*p* < 0.01, Figures 1F–H) compared with the HFD group. HE staining results revealed that the liver tissues were damaged with manifested extensive steatosis, cytoplasmic vacuolization, and inflammatory cell infiltration in HFD group by comparison with Control rats. YLZD could effectively reduce the lipid accumulation and inflammatory in the liver of NAFLD rats (Figure 1I). Moreover, ORO staining results showed that the liver tissues of HFD group rats were infiltrated with diffuse red lipid droplets, where these were larger in volume and partially fused into slices. Interestingly, the content and volume of lipid droplets were significantly decreased by YLZD intervention (Figure 1I). These results suggested that YLZD had a beneficial effect on the body weight, liver lipid accumulation and liver injury in the NAFLD rats induced by HFD.

### YLZD alleviated oxidative stress damage and reduced NAFLD-related systemic inflammation

**Figure 2:**
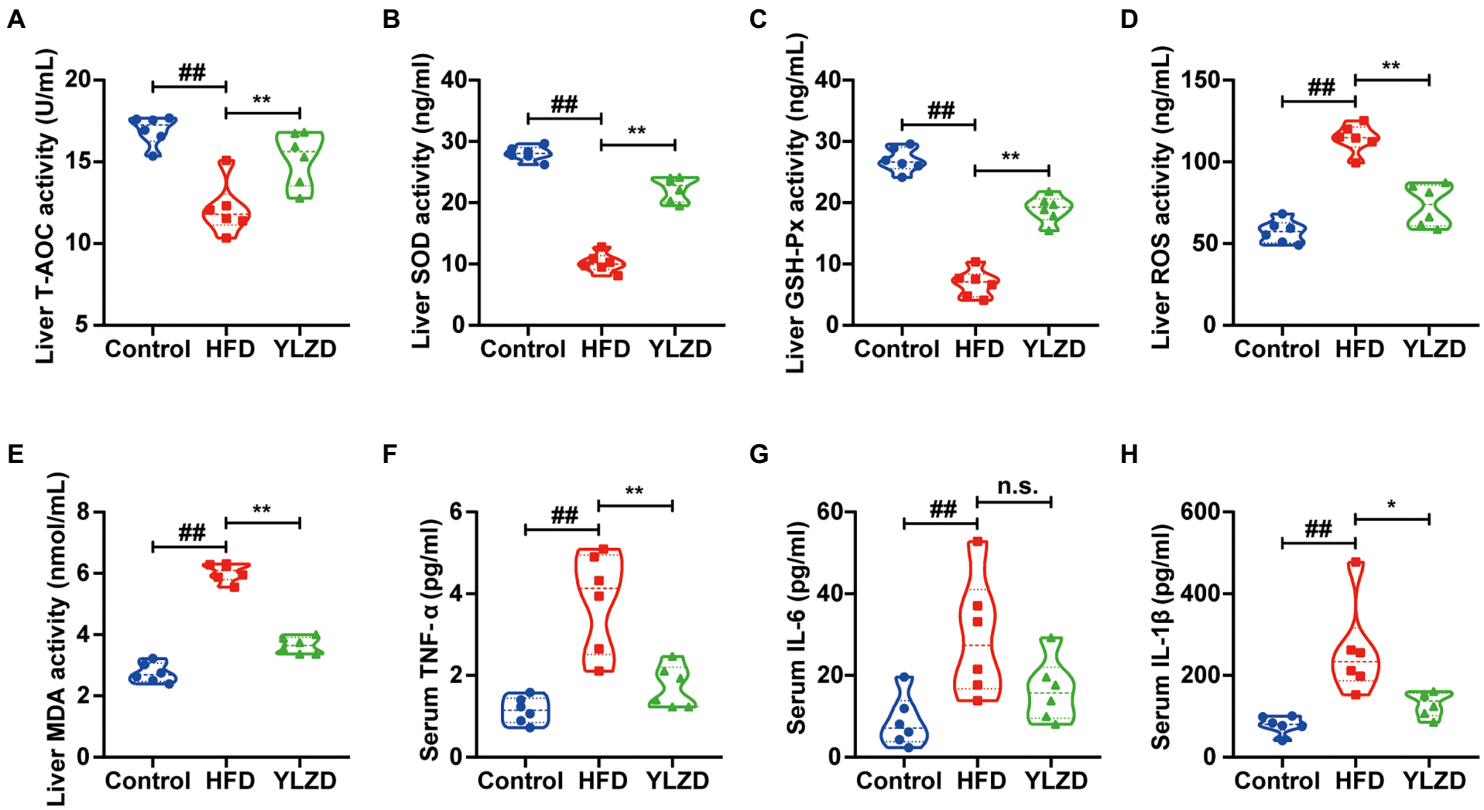
YLZD alleviated oxidative stress damage and reduced NAFLD-related systemic inflammation. Liver homogenate levels of **(A)** T-AOC, **(B)** SOD, **(C)** GSH-Px, **(D)** ROS and **(E)** MDA, respectively. Serum levels of **(F)** TNF-α, **(G)** IL-6 and **(H)** IL-1β, respectively. Values are represented as mean ± SEM (*n* = 6 rats/group). ^##^*p* < 0.01 versus the Control group; ^**^p < 0.01, ^*^p < 0.05 versus the HFD group.

Oxidative stress is the main pathogenesis of NAFLD, and reducing the oxidative stress level in the liver may bring new perspective for the prevention and treatment of NAFLD (Hong et al., 2021). The antioxidant enzymes (T-AOC, SOD and GSH-Px) and oxidation index (ROS and MDA) in the liver tissues of rats were measured to explicate the antioxidant effects of YLZD. As expected, the levels of T-AOC, SOD and GSH-Px in HFD group were significantly decreased (*p* < 0.01, Figures 2A–C), while the ROS and MDA levels were distinctly increased (*p* < 0.01, Figures 2D,E) compared with the Control group. Notably, YLZD treatment increased the T-AOC, SOD and GSH-Px levels and reduced the ROS and MDA levels to a varying degree by comparison with HFD group (*p* < 0.01, Figures 2A–E). The above results showed that YLZD may exert hepatoprotective effects by restoring the balance between oxidative and antioxidant systems.

Immune response is an important factor in the development and progression of NAFLD, and oxidative stress can trigger hepatic and systemic inflammatory responses (Manne et al., 2018; Arroyave-Ospina et al., 2021). In order to observe the inflammatory injury induced by a HFD, serum TNF-α, IL-6 and IL-1β levels were determined. The TNF-α, IL-6 and IL-1β levels in HFD group were markedly higher than those in Control group (*p* < 0.01, Figures 2F–H). YLZD intervention leads to a significant reduction in the TNF-α and IL-1β levels (*p* < 0.05 or 0.01, Figures 2F,H). There was no distinct difference in the level of IL-6 between the HFD group and the YLZD intervention group (Figure 2G). These data indicated that the YLZD intervention could effectively reduce the inflammatory response in the HFD-induced NAFLD rats.

### YLZD upregulated the expression levels of hepatic SIRT1/Nrf2 pathway-related genes and proteins

**Figure 3:**
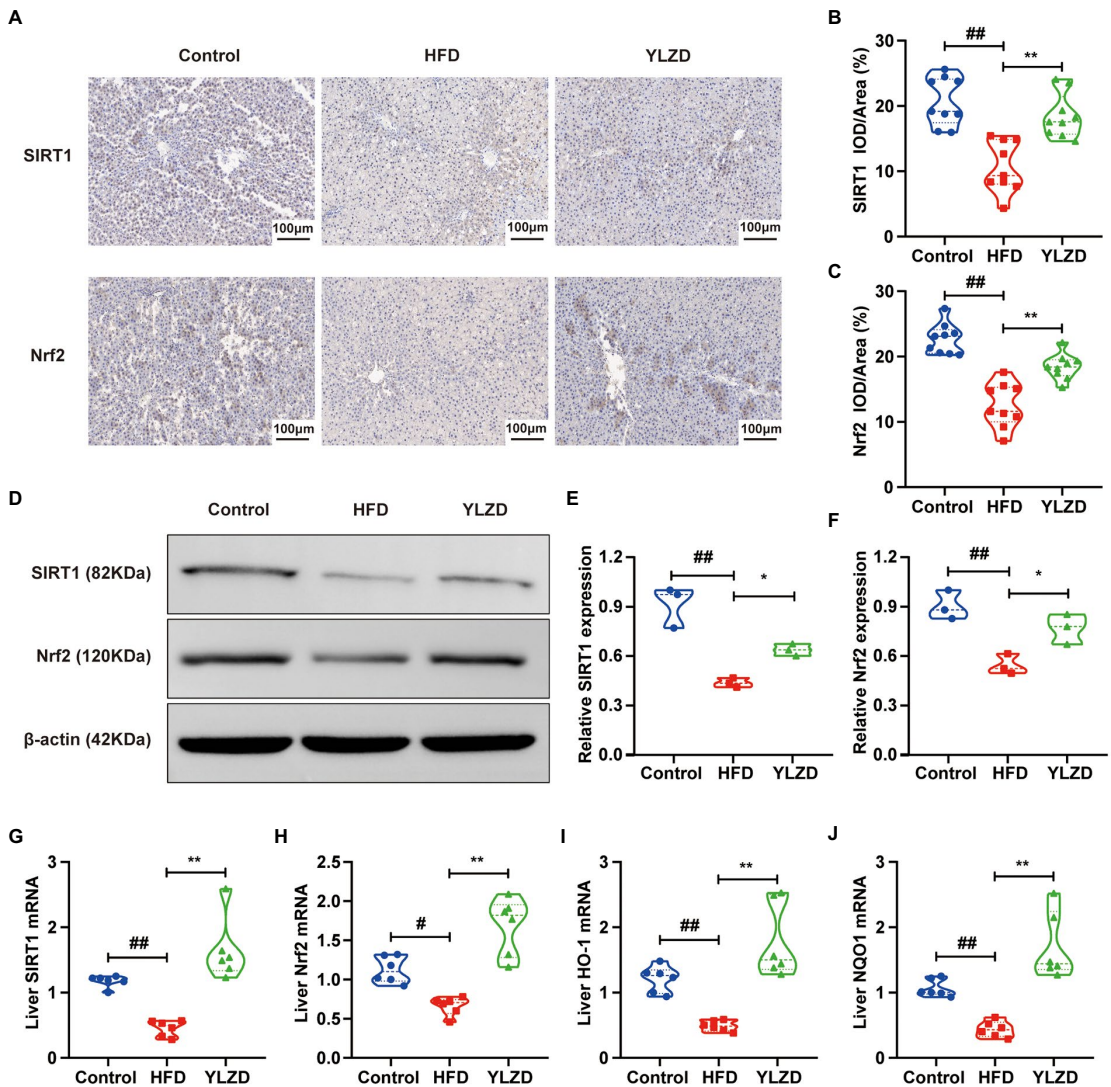
YLZD upregulated the expression levels of hepatic SIRT1/Nrf2 pathway-related genes and proteins. **(A)** Typical IHC staining images of the liver with different treatments (magnification, ×200). **(B,C)** Average optical density values (AOD = IOD/Area) of SIRT1 and Nrf2 protein expression. **(D)** Expression of transcription factors associated with SIRT1/Nrf2 pathway were detected by Western blot. **(E,F)** Quantification of the corresponding Western blotting results in D. **(G–J)** RT-qPCR of relative mRNA expression of *SIRT1*, *Nrf2*, *HO-1* and *NQO1* in liver. Values are represented as mean ± SEM (*n* = 6 rats/group). ^##^*p* < 0.01, ^#^*p* < 0.05 versus the Control group; ^**^p < 0.01, ^*^p < 0.05 versus the HFD group.

SIRT1 is considered to be a critical regulator involved in inflammation, apoptosis and antioxidant defense systems (Yang et al., 2016). Nrf2 is a primary transcription factor in the antioxidant response regulations (Sano et al., 2021). It has been reported that Nrf2 served as an important downstream target of SIRT1 signaling pathway, which increased the resistance to oxidative damage (Zhang et al., 2018). It is vital to explore whether the SIRT1/Nrf2 signaling pathway can regulate oxidative stress in NAFLD. Therefore, IHC, Western Blot and RT-qPCR methods were employed to further investigate the effect of YLZD on SIRT1/Nrf2 pathway and related factors. As shown in Figure 3A, the dark brown colored area representing the positive expression of SIRT1 and Nrf2 proteins in liver tissues of the Control group was mainly distributed in the cytoplasm around the confluent area-central vein. The expression of these two proteins in the HFD group was significantly reduced compared with the Control group. However, the YLZD intervention markedly increased the expression of SIRT1 and Nrf2 proteins with the range and color closer to the Control group. Mean optical density analysis demonstrated that the expression of SIRT1 and Nrf2 in rat hepatocytes was reduced in the HFD group by comparison with the Control group (*p* < 0.01, Figures 3B,C), and the expression of the proteins was substantially increased in the YLZD group than that in the HFD group (*p* < 0.01, Figures 3B,C). Western Blot analysis was adopted to further quantify the SIRT1 and Nrf2 proteins in liver tissues, confirming that YLZD could effectively increase the expression of SIRT1 and Nrf2 proteins (*p* < 0.05, Figures 3D–F). Furthermore, the expression levels of *SIRT1*, *Nrf2*, *HO-1* and *NQO-1* genes were detected by RT-qPCR method. The results revealed that the expression level of the above genes in the HFD group was reduced compared with the Control group (*p* < 0.05 or 0.01, Figures 3G–J), whereas YLZD intervention significantly promoted the expression of these genes (*p* < 0.01, Figures 3G–J). These results revealed that YLZD could activate the SIRT1/Nrf2 signaling pathway in the liver tissue of NAFLD rats.

### YLZD increased the concentration of serum SCFAs

**Figure 4:**
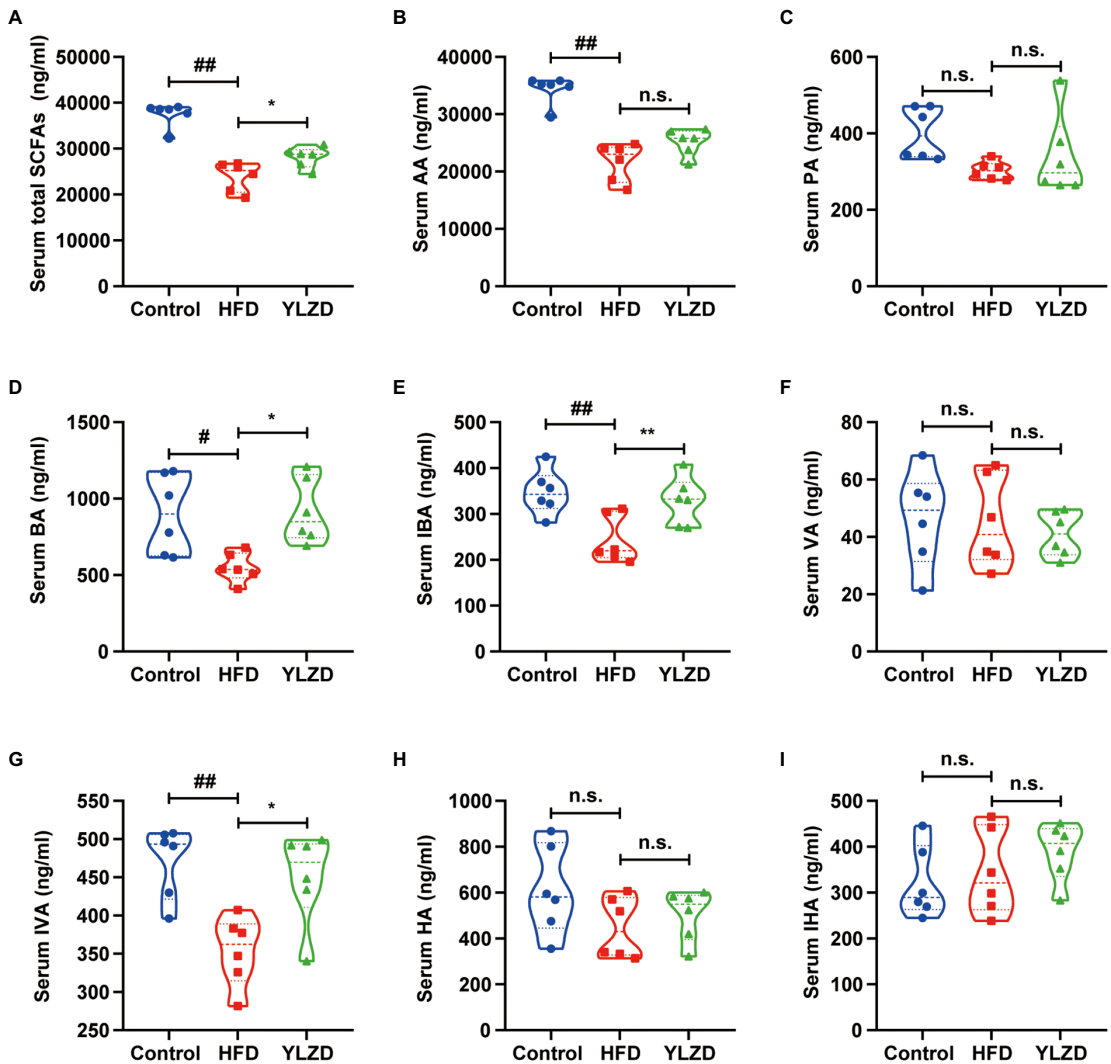
YLZD increased the concentration of serum SCFAs. **(A)** Serum total SCFAs, **(B)** serum AA, **(C)** serum PA, **(D)** serum BA, **(E)** serum IBA, **(F)** serum VA, **(G)** serum IVA, **(H)** serum HA, **(I)** serum IHA. AA, PA, BA, IBA, VA, IVA, HA and IHA are acetic acid, propionic acid, butyric acid, isobutyric acid, valeric acid, isovaleric acid, hexanoic acid and isohexanoic acid, respectively. Values are represented as mean ± SEM (*n* = 6 rats/group). ^##^*p* < 0.01, ^#^*p* < 0.05 versus the Control group; ^**^p < 0.01, ^*^p < 0.05 versus the HFD group.

SCFAs are produced in the gastrointestinal tract by intestinal bacteria, and those that are not metabolized in colonic cells enter the portal circulation of the liver through the basolateral membrane, which in turn affects host health (González-Bosch et al., 2021). It has been reported that SCFAs could prevent oxidative damage (Daschner et al., 2017), and specific SCFAs such as propionate together with butyrate are activators of the Keap1-Nrf2 defense pathway (Hoyles et al., 2018; Guo et al., 2020). The serum SCFAs concentrations were determined to investigate whether the hepatoprotective effect of YLZD is related to the concentration of SCFAs. Figure 4 depicted that the concentrations of AA, BA, IBA, IVA and total SCFAs were decreased by HFD exposure (*p* < 0.05 or 0.01). YLZD treatment remarkably increased the concentrations of BA, IBA, IVA and total SCFAs (*p* < 0.05 or 0.01) by comparison with that of HFD group. The above results indicated that the SCFAs concentrations were significantly reduced by HFD while this trend was largely reversed by YLZD intervention.

### YLZD raised the concentration of fecal SCFAs

**Figure 5:**
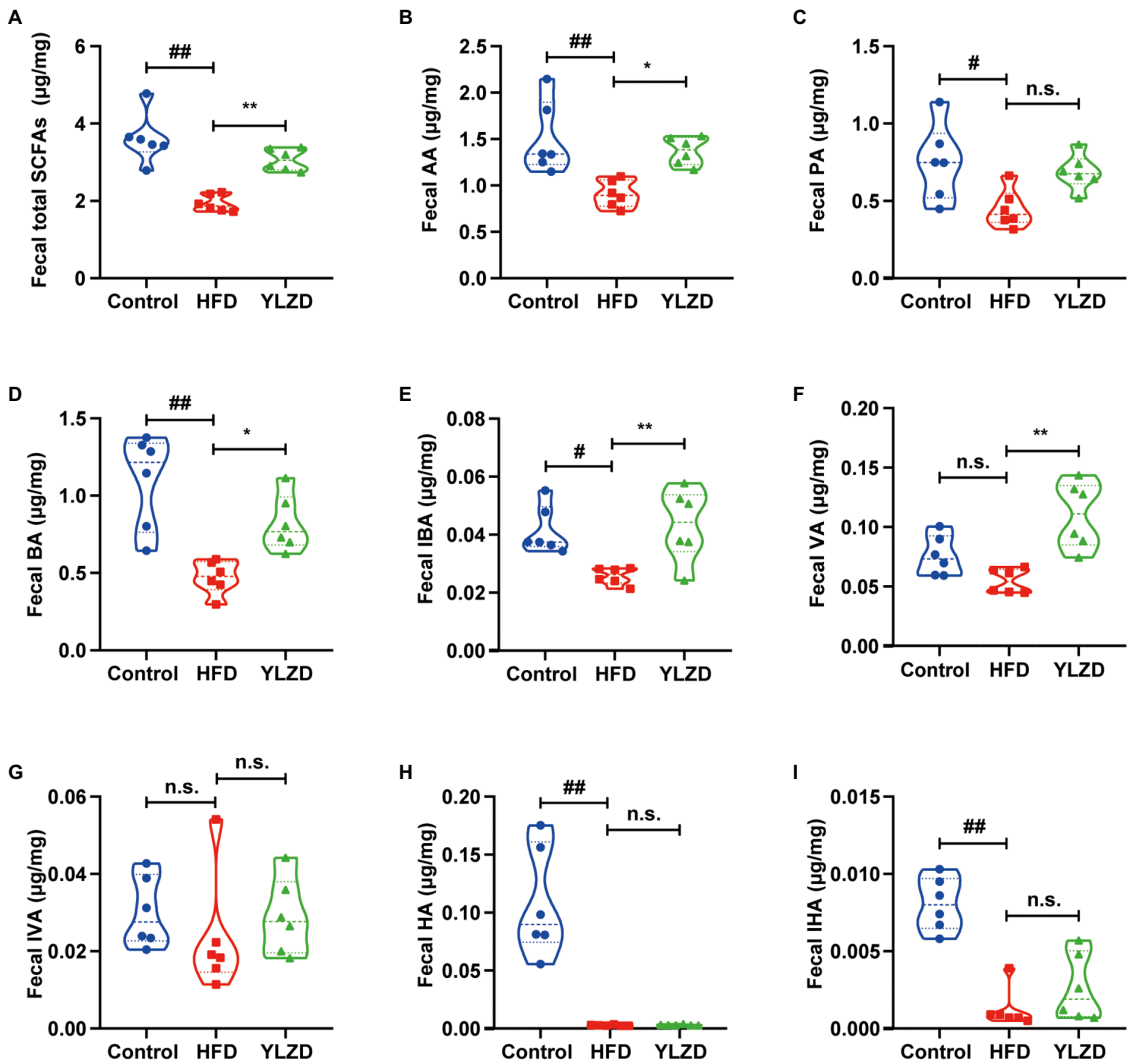
YLZD raised the concentration of fecal SCFAs. **(A)** Fecal total SCFAs, **(B)** fecal AA, **(C)** fecal PA, **(D)** fecal BA, **(E)** fecal IBA, **(F)** fecal VA, **(G)** fecal IVA, **(H)** fecal HA, **(I)** fecal IHA. Values are represented as mean ± SEM (*n* = 6 rats/group). ^##^*p* < 0.01, ^#^*p* < 0.05 versus the Control group; ^**^p < 0.01, ^*^p < 0.05 versus the HFD group.

Some of the SCFAs in the intestine are absorbed by the enterocytes to produce energy; some are released through the basolateral membrane of the enterocytes to the hepatic portal vein and enter the circulation *via* the liver while the remaining unabsorbed SCFAs are excreted in the feces (Zhang et al., 2021). Therefore, the concentration of SCFAs in feces can reflect their concentrations in the intestine. Consequently, the GC–MS method was employed to further illustrate the effect of YLZD on the SCFAs concentrations in the feces of HFD rats. Figure 5 shows that the concentrations of AA, PA, BA, IBA, HA, IHA and total fecal SCFAs in HFD rats were obviously lower than Control group (*p* < 0.05 or 0.01). Interestingly, YLZD administration upregulated the concentrations of AA, BA, IBA, VA and total SCFAs compared with HFD group (*p* < 0.05 or 0.01). These results demonstrated that YLZD could increase the concentrations of SCFAs, the intestinal metabolites of HFD-induced NAFLD rats.

### YLZD remodeled the composition of gut microbiota

**Figure 6:**
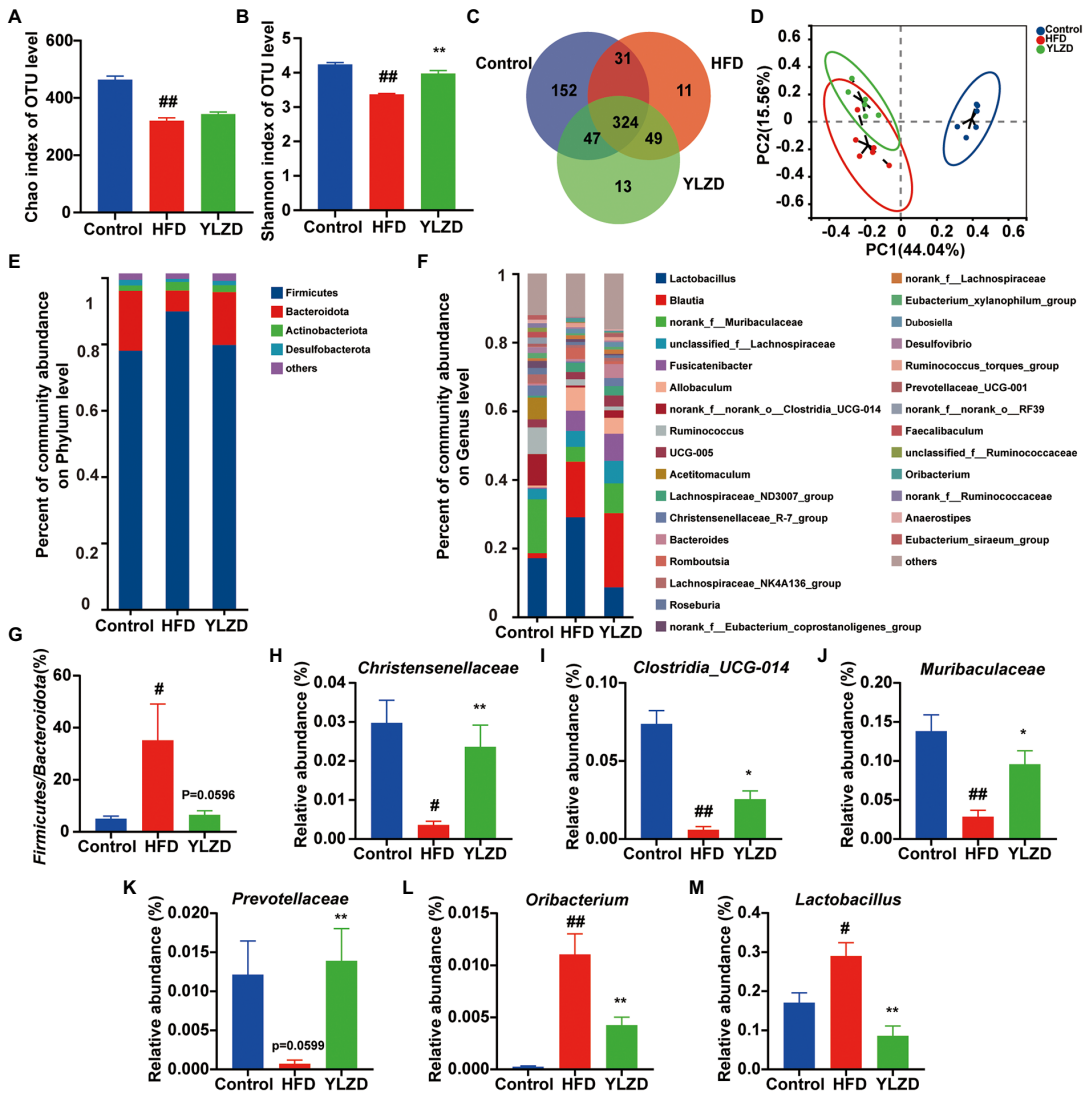
YLZD remodeled the composition of gut microbiota. **(A,B)** The bacterial diversity in intestinal estimated by alpha-diversity of chao and Shannon indexes. **(C)** Venn diagram of the overlap of the OTUs in the gut microbiota in different treatments. **(D)** The β-diversity analysis of Principal Coordinate Analysis (PCoA). **(E)** Bar plot of the gut microbiota at the phylum level. **(F)** Bar plot of the gut microbiota at the genus level. **(G)** The ratio of *Firmicutes* to *Bacteroidetes*. **(H–M)** Changes in the abundance of *Christensenellaceae*, *Clostridia_UCG-014*, *Muribaculaceae*, *Prevotellaceae*, *Oribacterium* and *Lactobacillus*, respectively. Values are represented as mean ± SEM (*n* = 6 rats/group). ^##^*p* < 0.01, ^#^*p* < 0.05 versus the Control group; ^**^p < 0.01, ^*^p < 0.05 versus the HFD group.

Gut microbiota is of great significance for maintaining intestinal homeostasis and human health. Gut microbiota imbalance has been reported to be closely associated with the development of NAFLD (Wang et al., 2021). Previous research also suggested that the content of SCFAs was closely related to the composition of gut microbiota (Koh et al., 2016). Therefore, the high-throughput sequencing of 16S rRNA was used to analyze rat fecal samples for further illustrating the correlation between changes in SCFAs and gut microbiota in HFD-induced NAFLD rats. After screening and quality control, a total of 1,053,670 optimized sequences were obtained from fecal samples of 18 rats (6 each from Control, HFD and YLZD groups). In addition, 627 OTUs, including 9 phyla, 291 species and 167 genera of intestinal microorganisms, were matched and annotated for subsequent analysis. Alpha diversity was analyzed through calculating Chao and Shannon in OTU level (Figures 6A,B). The results indicated that the microbial diversity of the fecal microbiome was decreased in HFD group rats (*p* < 0.01) while it was incremental increased by YLZD intervention (*p* < 0.01). Venn diagram (Figure 6C) analysis showed that the number of OTUs shared by the three groups was 324, and the number of OUTs shared by the Control and HFD, Control and YLZD, HFD and YLZD groups were 355, 371, and 373, respectively. PCoA analysis (Figure 6D) indicated that there existed a clear separation between the Control and HFD group, illustrating that the structure of gut microbiota can be changed by HFD. However, the gut microbiota of NAFLD rats was more inclined to Control group rats after YLZD intervention. In summary, gut microbiota diversity was significantly reduced in HFD-induced NAFLD rats, and YLZD could modulate the gut microbiota distribution and restore it closer to the healthy state.

To identify the changes in the gut microbial community, bar graphs at the phylum and genus level were performed for all groups. The microbial community structure was dominated by the *Firmicutes* and *Bacteroides* at the phylum level (Figure 6E). Furthermore, the richness of *Firmicutes* in HFD group was higher than that in Control group, while it was lower for the *Bacteroides*, and YLZD reversed the change of *Bacteroides* (*p* < 0.05, Supplementary Figure S2). Besides, the *Firmicutes/Bacteroides* ratio in the HFD group was markedly higher than for the Control group (*p* < 0.05), and the ratio was reduced by YLZD treatment (*p* = 0.0596, Figure 6G). At the genus level, HFD feeding reduced *Christensenellaceae*, *Clostridia_UCG-014*, *Muribaculaceae* and *Prevotellaceae*, enlarged *Oribacterium* and *Lactobacillus* (*p* < 0.05 or 0.01, Figures 6F,H–M). Notably, YLZD could partly reverse these changes (*p* < 0.05 or 0.01, Figures 6F,H–M). These data demonstrated the modulatory effect of YLZD on the gut microbiota of HFD-induced NAFLD rats.

### Correlation between indicators of liver injury, oxidative stress-related enzymes, SCFAs levels and gut microbiota composition changes

**Figure 7:**
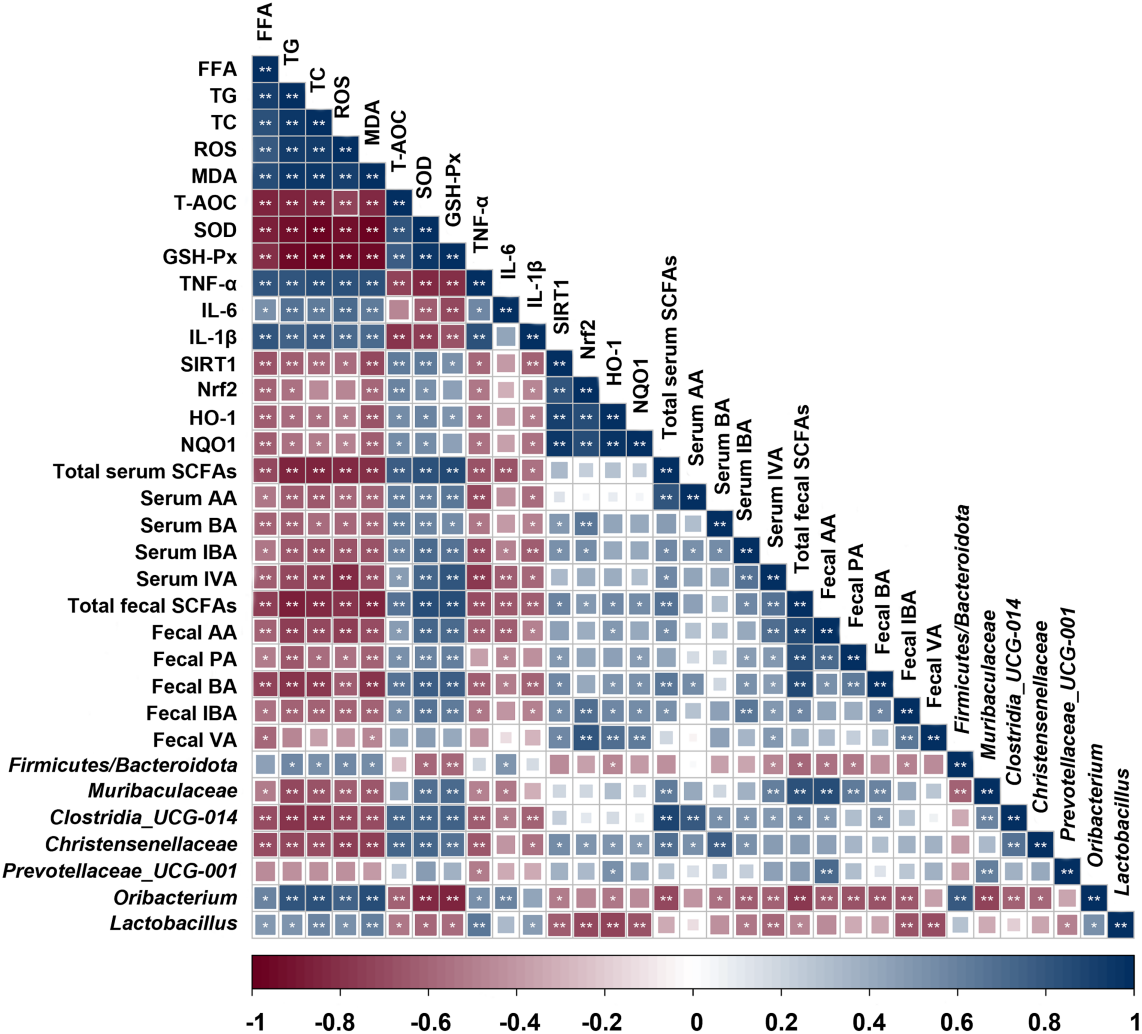
Correlation between indicators of liver injury, oxidative stress-related enzymes, SCFAs levels and gut microbiota composition changes. Blue indicates positive correlation and red indicates negative correlation. ^**^p < 0.01, ^*^p < 0.05.

Spearman rank correlation analysis was employed to explore the correlation between indicators of liver injury, oxidative stress-related enzymes, SCFAs levels and gut microbiota composition changes (Figure 7). The results showed that the probiotics (*Christensenellaceae*, *Clostridia_UCG-014*, *Muribaculaceae* and *Prevotellaceae*) presented a negative correlation with the liver lipid levels (FFA, TG and TC; *p* < 0.05 or 0.01), lipid peroxidation product MDA, and inflammatory factors (TNF-α, IL-6, and IL-1β; *p* < 0.05 or 0.01). Furthermore, these probiotics were found to be significant positively correlated with liver antioxidant enzymes (SOD and GSH-Px; *p* < 0.01). However, *Oribacterium* and *Lactobacillus* were completely distinct from the above probiotics and exerted an opposite effect. Additionally, the serum and fecal SCFAs levels represented a negative correlation with liver lipid and inflammatory factors levels, while they were positively correlated with liver antioxidant enzymes. Therefore, these results supported that different microbiota profiles and SCFAs distinctly influenced the expression of liver factors related to lipid metabolism and oxidative damage.

### Effects of acetic acid and BA on cell viability and FFA-induced lipid accumulation and oxidative stress in L02 cells

**Figure 8:**
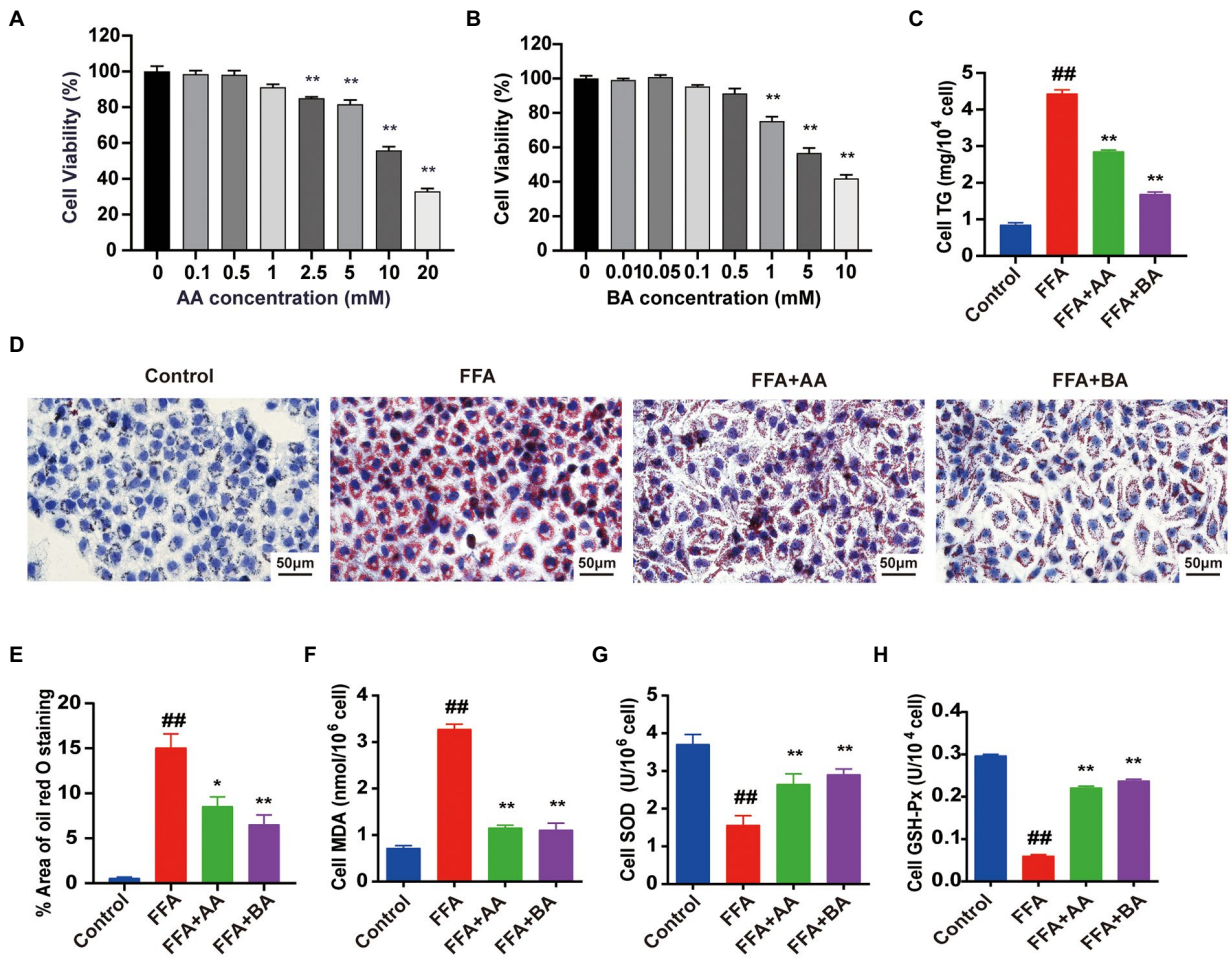
Effects of acetic acid and butyric acid on cell viability and FFA-induced lipid accumulation and oxidative stress in L02 cells. **(A,B)** The effects of AA and BA on L02 cell viability performed by CCK8 assays. **(C)** Cell TG level. **(D)** Cell Oil red O staining (magnification, ×400). **(E)** Quantitative analysis of oil red staining results was performed using Image Pro Plus 6.0 software. **(F–H)** The level of cell MDA, SOD and GSH-Px, respectively. L02 cells were pre-treated for 24 h with 1.0 mM FFA to constructed NAFLD cell model before treated with 1.0 mM AA or 0.5 mM BA for another 24 h. Data represent means ± SEM. ^##^*p* < 0.01 versus Control; ^*^p < 0.05, ^**^p < 0.01 versus FFA.

SCFAs, mainly including AA, PA and BA, are not only metabolic substrates, but also exerting positive therapeutic effects in human-related diseases (Liu et al., 2021). Our previous *in vivo* researches confirmed that YLZD treatment significantly increased the SCFAs contents in serum and fecal in NAFLD rats, especially the AA and BA levels. In order to further illustrate the antioxidant effect of AA and BA *in vitro*, L02 cells were pre-treated for 24 h with 1.0 mM FFA (the best concentration and timing of mold making that have been determined, Supplementary Figure S3) to construct NAFLD cell model before the treatment with AA or BA for another 24 h. To evaluate the cytotoxic effect of AA, BA and identify a suitable concentration for subsequent L02 cells experiments, CCK8 assays were performed. The results indicated that AA (0–1 mM) and BA (0–0.5 mM) exerted no conspicuous inhibitory effect on the growth of L02 cells (Figures 8A,B) and thus 1 mM AA and 0.5 mM BA were chosen ultimately. The TG levels and ORO staining results showed that the supplementation of AA and BA dramatically reduced the cellular lipid deposit (*p* < 0.05 or 0.01, Figures 8C–E). Then, the antioxidation effect of AA and BA on FFA-induced L02 cells was investigated, demonstrating that AA and BA reversed the increased MDA formation and the reduction of SOD and GSH-Px levels caused by FFA (*p* < 0.01, Figures 8F–H). The results indicated that AA and BA attenuated FFA-induced cell lipid accumulation and oxidative stress, and BA generated the more potent efficacy.

### BA ameliorated FFA-induced L02 cells oxidative stress injury *via* the SIRT1/Nrf2 pathway

**Figure 9:**
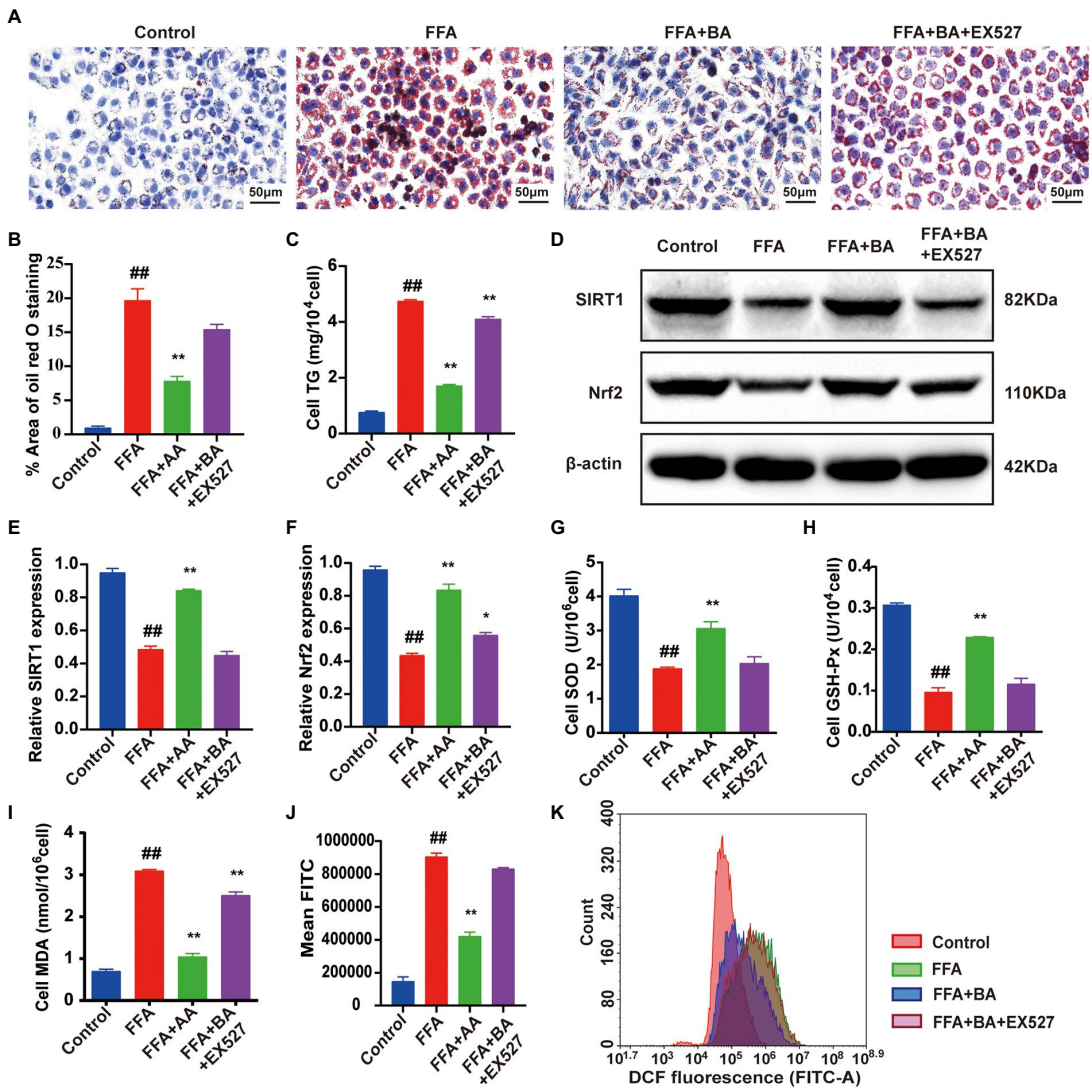
Butyric acid ameliorated FFA-induced L02 cells oxidative stress injury *via* the SIRT1/Nrf2 pathway. **(A)** Cell Oil red O staining (magnification, ×400). **(B)** Quantitative analysis of oil red staining results was performed using Image Pro Plus 6.0 software. **(C)** Cell TG level. **(D–F)** Western blot analysis of SIRT1 and Nrf2 in L02 cells. **(G–I)** The level of cell SOD, GSH-Px and MDA, respectively. **(J,K)** Histogram of intracellular ROS level detected by DCFH-DA. L02 cells were pre-treated for 24 h with 1.0 mM FFA to constructed NAFLD cell model before treated with 0.5 mM BA or 100 nM EX-527 for another 24 h. Data represent means ± SEM. ^##^*p* < 0.01 versus Control; ^*^p < 0.05, ^**^p < 0.01 versus FFA.

To illustrate BA ameliorated lipid accumulation and oxidative stress in FFA-induced L02 cells *via* the regulation of SIRT1/Nrf2-dependent signaling, L02 cells were further treated with EX-527 (100 nM), which is an SIRT1 inhibitor, for 24 h. CCK8 was used to screen the optimal concentrations of EX-527 (Supplementary Figure S4). BA-supplementation strongly decreased the lipid accumulation compared with the model group (*p* < 0.01), whereas this could be restricted by EX-527 (*p* < 0.01, Figures 9A–C). Furthermore, the protein expression of SIRT1 and Nrf2 was significantly decreased in FFA-induced L02 cells contrast to the control group (*p* < 0.01, Figures 9D–F). Though the SIRT1 and Nrf2 expressions were dramatically elevated by the supplementation of BA (*p* < 0.01), the effect of BA could be restrained by EX-527 (*p* < 0.05, Figures 9D–F). In addition, EX-527 partly abolished the up-regulative effect of BA on SIRT1/Nrf2 pathway downstream antioxidant factors such as SOD and GSH-Px (*p* < 0.01, Figures 9G,H), blocked the down-regulation of ROS and MDA produced by BA in FFA-induced L02 cells (*p* < 0.01, Figures 9I–K). Taken together, these results showed that BA can trigger SIRT1/Nrf2 signal transduction, leading to the upregulation of downstream antioxidative factors of SIRT1/Nrf2 pathway, thereby suppressing FFA-induced oxidative stress.

## Discussion

NAFLD is a complex and systemic metabolic disease with an increasing incidence worldwide. Chinese herbs and compound medicine, show great potential. YLZD may be conducive to the NAFLD interventions due to its potential lipid regulation and antioxidant activities (Guo et al., 2017). The current study found that YLZD reduced body weight, liver index and epididymal fat index in NAFLD rats, which were not achieved by restricting energy intake in rats. We speculate that YLZD may decrease the body weight of NAFLD rats by reducing visceral fat deposition and promoting lipid excretion, which was supported by liver FFA, TG, and TC results in NAFLD rats. In addition, YLZD can improve liver function in NAFLD rats, significantly reducing AST and ALT levels. Our previous study (Jiang et al., 2022) found that the improvement was more pronounced with increasing the dose of YLZD administration, and no obvious hepatotoxic effects were observed. It follows that YLZD has an effective therapeutic effect on NAFLD and its potential intervention mechanism is worthy of further investigation.

Oxidative stress, characterized by an imbalance between antioxidant enzyme systems and pro-oxidant responses, is closely correlated with the NAFLD progression (Gopinath et al., 2021). It is critical to promote a balance between oxidative and antioxidant responses to prevent the transition from NAFLD to NASH. SIRT1 plays a vital role as a deacetylase in biological processes such as oxidative stress, apoptosis and senescence, gene transcription, and metabolism (Carafa et al., 2016). It has been extensively studied in liver diseases in recent years. Bu et al. (2017) found that the activation of SIRT1 significantly inhibited the production of ROS and attenuated oxidative stress and hepatocyte injury. Zhang et al. (2017) reported that resveratrol, an important natural polyphenol antioxidant, could activate the SIRT1 signaling pathway to participate in the antioxidant response of cells. Even more significantly, SIRT1 is involved in the regulation of multiple stimulus-related oxidative stress by the activation of Nrf2, suggesting that Nrf2 might be an important downstream target of SIRT1 signaling pathway (Shi et al., 2019). Nrf2 is a key regulator of antioxidant signaling, and is the most crucial signaling pathway for cellular defense against oxidative stress (Zakaria et al., 2021). Moreover, it was documented that Nrf2 was dissociated from Keap1 and translocated into the nucleus under high oxidative stress conditions, ultimately activating the expression of the ARE, stimulating the transcriptions of various anti-oxidative enzyme genes including SOD, GSH-Px, HO-1, NQO1, and protecting the body from various oxidative stress damages (Kensler et al., 2007; Xu et al., 2019; Han et al., 2020). In addition, related research had found that Nrf2 activation significantly improved hepatic steatosis and inflammatory response in NAFLD mice (Xia et al., 2018; Shen et al., 2020), while the knockdown of Nrf2 gene exacerbated the disrupted glycolipid metabolism and inflammatory response in HFD exposed mice (Meakin et al., 2014; Liu et al., 2016). HO-1 and NQO-1 are downstream target genes of Nrf2, where these are important enzymes for scavenging free radicals in organisms and can alleviate cell membrane lipid peroxidation (Zhang et al., 2021). In this study, YLZD treatment upregulates the expression of SIRT1, Nrf2 and improves the activities of anti-oxidative factors such as NQO-1, HO-1, SOD and GSH-Px in HFD-induced NAFLD rats. The results indicated that the NAFLD-related oxidative stress injury could be markedly improved by YLZD through increasing the antioxidize enzyme activities and inhibiting the oxidation factor activities in the liver.

The gut microbiota is a complex and stable microecological environment which is of great importance for maintaining human health (Aron-Wisnewsky et al., 2020; Chen and Vitetta, 2020). It has been shown that dysbiosis of gut microbiota may induce NAFLD and obesity associated with oxidative stress (Borrelli et al., 2018; He et al., 2021). In the current research, the structure of gut microbiota of the HFD-induced NAFLD rats changed. The 16S rRNA high-throughput sequencing results showed that YLZD treatment increased the OTU number, upregulated Shannon index, and reversed the decrease of bacterial diversity in NAFLD rats. PCoA results revealed that YLZD intervention restored the gut microbiota composition of NAFLD rats close to that of healthy rats. The *Firmicutes*/*Bacteroides* ratio is an important factor to distinguish NAFLD patients from healthy individuals and reducing this ratio is conducive to the improvement of NAFLD (Ley et al., 2005; Lu et al., 2019). In this study, the relative abundance of *Firmicutes* in the intestine of HFD induced NAFLD rats increased compared with the control group, and the abundance of *Bacteroides* decreased, leading to a significant increase in the ratio of *Firmicutes*/*Bacteroides*. YLZD treatment markedly reversed the *Firmicutes*/*Bacteroides* ratio. In addition, at the genus level, the proportion of *Lactobacillus* and *Oribacterium* in the feces of HFD-induced NAFLD rats increased, while the proportion of *Christensenellacea*, *Clostridia*, *Muribaculateae* and *Prevotellariae* decreased. Notably, the proportion of the above bacteria was reversed by YLZD intervention. *Oribacterium* is closely related to adipogenesis and has been reported to be positively correlated with the expression of adipogenic gene CD36 (Petrov et al., 2019). Generally, *Lactobacillus* had a positive effect on metabolic disorders such as NAFLD since it was a probiotic, and HFD would reduce the *Lactobacillus* content in the intestine of rats (Liu et al., 2021). However, some studies found that *Lactobacillus* was increased in NAFLD patients and HFD-induced NAFLD rats (Raman et al., 2013; Bai et al., 2019; Ding et al., 2020), which is consistent with our results, but the specific reasons for this phenomenon need to be further investigated. *Christensenellaceae* is a recently discovered family of *Firmicutes* with important implications for human health (Waters and Ley, 2019). *Christensenellaceae*, which was reported to be the major producers of SCFAs, was found at significant lower levels in the gut for patients with metabolic syndrome (MetS) and obesity (Waters and Ley, 2019; Jing et al., 2021). *Clostridia*, a group of anaerobic bacteria under the *Firmicutes*, were enriched in the intestine of healthy rats and could prevent the HFD-induced weight gain in rats through blocking the ability of the intestine to absorb fat (Petersen et al., 2019). Besides, *Muribaculaceae* is the main producer of acetate and propionate (Ormerod et al., 2016). *Prevotellaceae* family contributes to the breakdown of carbohydrates from dietary fiber and produces SCFAs, which regulates the activity of the enteric nervous system and is conductive to the maintenance of intestinal homeostasis (Unger et al., 2016). In summary, YLZD might treat NAFLD through regulating the gut microbiota and increasing the content of SCFAs in intestinal by enriching some of the beneficial SCFAs-producing bacteria.

SCFAs are carboxylic acids with aliphatic tails of 1–6 carbons. It is the main products of intestinal microbial fermentation and derived from indigestible carbohydrates in the colon (Koh et al., 2016; Parada Venegas et al., 2019). Some studies have reported that the changes in SCFAs content were closely related to the formation of oxidative stress and inflammatory microenvironment in the pathogenesis of NASH (Zhou and Fan, 2019; Deng et al., 2020). Multiple evidences suggested that SIRT1 was activated by SCFAs directly or indirectly (Jiao et al., 2020). In addition, specific SCFAs (such as BA and PA) are activators of the Keap1-Nrf2 defense pathway, which can activate cellular antioxidant mechanisms and downregulate the expression of pro-inflammatory factors (Guo et al., 2020; Chen et al., 2021; González-Bosch et al., 2021). It is evident that SCFAs could link the microbiota with the maintenance of host redox homeostasis *via* SIRT1/Nrf2 signaling. However, previous studies mainly focused on the analyses of SCFAs from feces. In the present study, the SCFAs levels in the serum were measured. We found that the content of BA, IBA, and IVA in the serum of HFD rats decreased significantly, while YLZD intervention reversed this trend. Furthermore, the correlation between the gut microbiome, SCFAs and SIRT1/Nrf2 signaling pathway was further analyzed using a Spearman correlation matrix. A strong correlation between gut microbiota and SCFAs was found, especially the close correlation between *Christensenellaceae* and AA, BA, together with total SCFAs, and the strong correlation between SCFAs such as BA with SIRT1/Nrf2 pathway. *In vitro*, our research confirmed that AA and BA reduced FFA-induced lipid deposition and oxidative stress in L02 cells. Additionally, BA could activate the SIRT1/Nrf2 signaling pathway and increase the expression of antioxidant factors downstream of this pathway, which in turn ameliorated oxidative stress.

In conclusion, YLZD exerts a therapeutical effect on HFD-induced NAFLD by modulation of SIRT1/Nrf2 signaling pathway, which corresponds with shaping gut microbiota, increasing the content of SCFAs-producing bacteria such as *Christensenellaceae*, and upregulating SCFAs such as BA levels in NAFLD rats. Moreover, *in vitro* study confirmed that BA effectively reduced oxidative stress by activating SIRT1/Nrf2 signaling pathway in L02 cells. Our results provide new insights into the mechanism of YLZD for the treatment of NAFLD, and hold promise for the botanical-based therapies in NAFLD.

## Data availability statement

The datasets presented in this study can be found in online repositories. The names of the repository/repositories and accession number(s) can be found in the article/Supplementary material.

## Ethics statement

The animal study was reviewed and approved by Animal Research Ethics Committee of Beijing University of Chinese Medicine. Written informed consent was obtained from the owners for the participation of their animals in this study.

## Author contributions

HH and JL conceived and designed the study. HJ completed the experiments, organize and analyze microbiota data, wrote the first draft, and substantially revised the manuscript. ZS, LS, XH, YZ, XZ, JW, JH, and LZ provided various experimental assistance, such as specimen collection and statistical analysis. TM critically revised the manuscript, supervised and verified the data from this experiment. All authors contributed to the article and approved the submitted version.

## Funding

This work was supported by Beijing Natural Science Foundation (no. 7202124), National Science Foundation of China (no. 82074344), and the Innovation “One Hundred Million” Talent Project Qihuang Scholar.

## Conflict of interest

The authors declare that the research was conducted in the absence of any commercial or financial relationships that could be construed as a potential conflict of interest.

## Publisher’s note

All claims expressed in this article are solely those of the authors and do not necessarily represent those of their affiliated organizations, or those of the publisher, the editors and the reviewers. Any product that may be evaluated in this article, or claim that may be made by its manufacturer, is not guaranteed or endorsed by the publisher.

## Supplementary material

The Supplementary material for this article can be found online at: https://www.frontiersin.org/articles/10.3389/fmicb.2022.1001778/full#supplementary-material
