# Supplementary material for: Yinchen Linggui Zhugan decoction ameliorates high fat diet-induced nonalcoholic fatty liver disease by modulation of SIRT1/Nrf2 signaling pathway and gut microbiota
Source: Front Microbiol. 2022 Dec 12;13:1001778. doi: 10.3389/fmicb.2022.1001778 (PMC9791106; doi:10.3389/fmicb.2022.1001778)

Supplementary Figure

**Supplementary Figure 1.** Model validation-HE staining (200 ×).


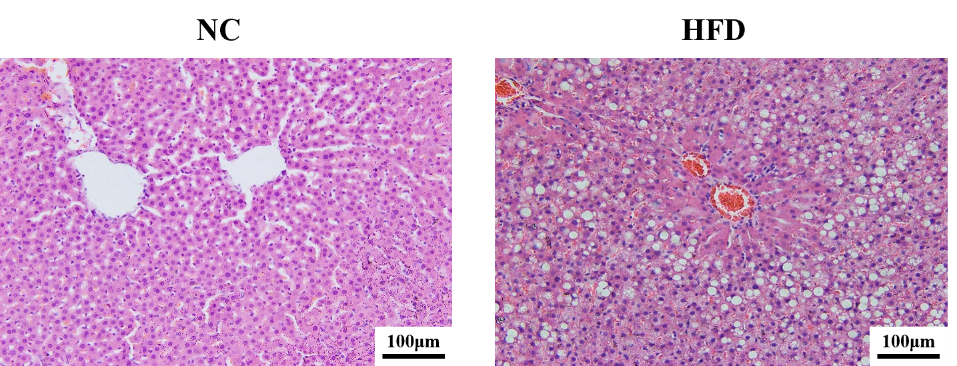


**Supplementary Figure 2.** Relative abundance of *Firmicutes* and *Bacteroidetes*.


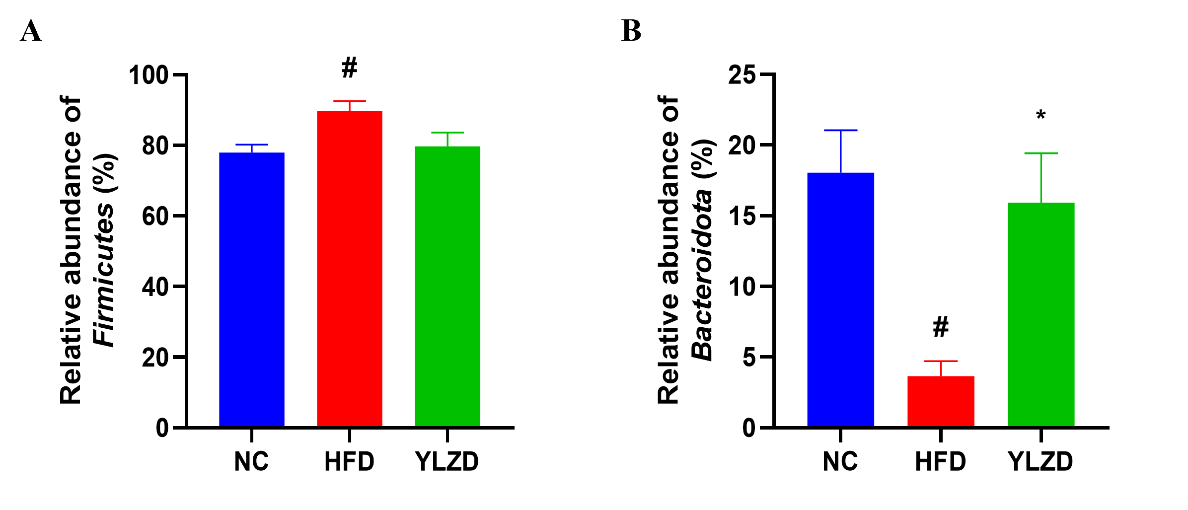


**Supplementary Figure 3.** The best intervention concentration and time of FFA was screened by CCK8 assays.


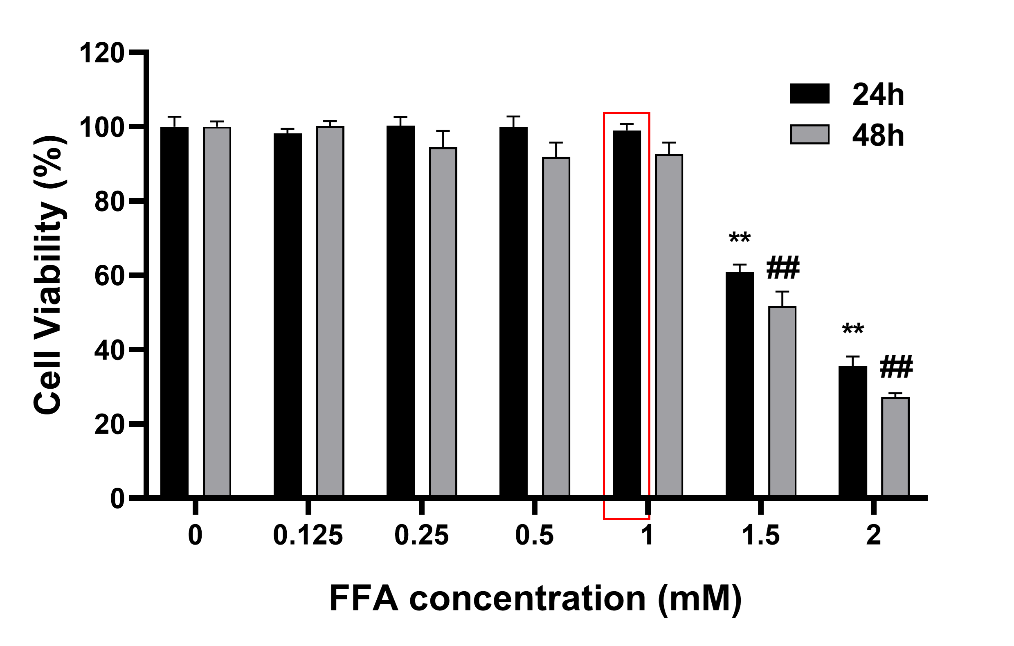


**Supplementary Figure 4.** The best intervention concentration of EX-527 was screened by CCK8 assays.


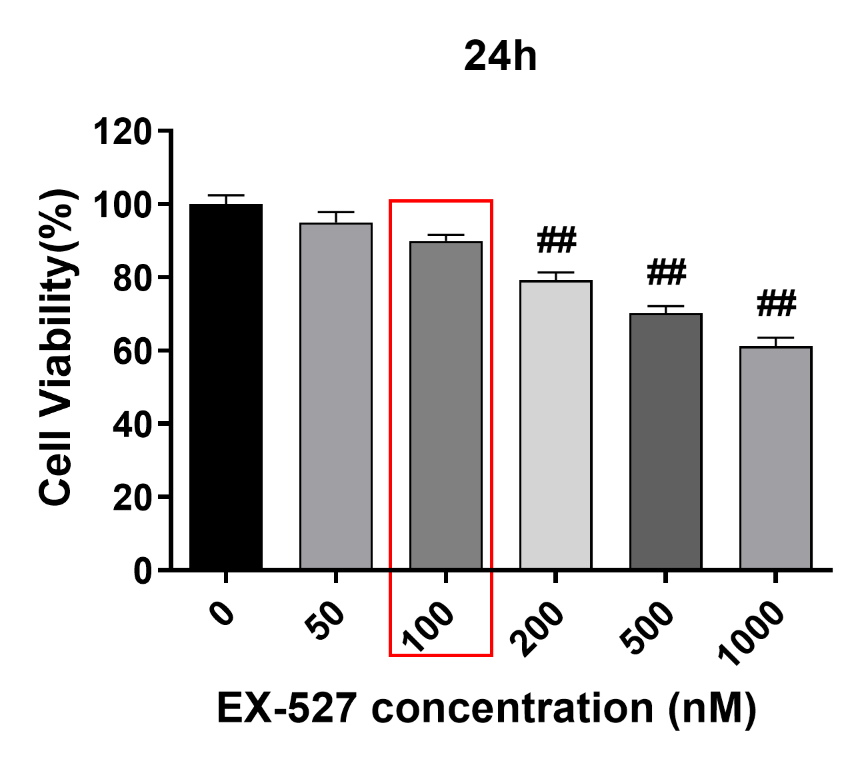

Supplement: Supplementary file 1 [file Data_Sheet_1.docx]
